# Diagnostic and Prognostic Value of pH- and Oxygen-Sensitive Magnetic Resonance Imaging in Glioma: A Retrospective Study

**DOI:** 10.3390/cancers14102520

**Published:** 2022-05-20

**Authors:** Jingwen Yao, Akifumi Hagiwara, Talia C. Oughourlian, Chencai Wang, Catalina Raymond, Whitney B. Pope, Noriko Salamon, Albert Lai, Matthew Ji, Phioanh L. Nghiemphu, Linda M. Liau, Timothy F. Cloughesy, Benjamin M. Ellingson

**Affiliations:** 1 UCLA Brain Tumor Imaging Laboratory (BTIL), Center for Computer Vision and Imaging Biomarkers, University of California, Los Angeles, CA 90024, USA; jingwen.yao@ucsf.edu (J.Y.); hagiwara.akifumi.0314@gmail.com (A.H.); toughourlian@mednet.ucla.edu (T.C.O.); chencaiwang@mednet.ucla.edu (C.W.); craymond@mednet.ucla.edu (C.R.); 2 Department of Radiological Sciences, David Geffen School of Medicine, University of California, Los Angeles, CA 90024, USA; wpope@mednet.ucla.edu (W.B.P.); nsalamon@mednet.ucla.edu (N.S.); 3 Neuroscience Interdepartmental Program, David Geffen School of Medicine, University of California, Los Angeles, CA 90024, USA; 4 UCLA Neuro-Oncology Program, University of California, Los Angeles, CA 90024, USA; albertlai@mednet.ucla.edu (A.L.); matthewji12345@ucla.edu (M.J.); pnghiemphu@mednet.ucla.edu (P.L.N.); tcloughesy@mednet.ucla.edu (T.F.C.); 5 Department of Neurology, David Geffen School of Medicine, University of California, Los Angeles, CA 90024, USA; 6 Department of Neurosurgery, David Geffen School of Medicine, University of California, Los Angeles, CA 90024, USA; lliau@mednet.ucla.edu

**Keywords:** multiparametric MRI, glioma, survival analysis, genotype, tumor microenvironment

## Abstract

**Simple Summary:**

Gliomas are known to present with an altered metabolic phenotype that contributes to the abnormal tumor microenvironment detectable on MRI. The aim of this study was to quantify metabolic statuses of glioma using pH- and oxygen-sensitive MRI and associate the measurements with genetic mutation and prognosis. Using the data of 159 adult glioma patients, we revealed that isocitrate dehydrogenase mutation, 1p/19q co-deletion, and epidermal growth factor receptor amplification statuses were associated with the MRI measurements revealing tissue acidosis and hypoxia, and these measurements were also associated with progression-free survival and overall survival, independent of patient age, treatment status, and isocitrate dehydrogenase mutation status. In conclusion, the pH- and oxygen-sensitive MRI is clinically feasible and potentially valuable for distinguishing glioma genotypes and provides additional prognostic value to clinical practice.

**Abstract:**

Characterization of hypoxia and tissue acidosis could advance the understanding of glioma biology and improve patient management. In this study, we evaluated the ability of a pH- and oxygen-sensitive magnetic resonance imaging (MRI) technique to differentiate glioma genotypes, including isocitrate dehydrogenase (IDH) mutation, 1p/19q co-deletion, and epidermal growth factor receptor (EGFR) amplification, and investigated its prognostic value. A total of 159 adult glioma patients were scanned with pH- and oxygen-sensitive MRI at 3T. We quantified the pH-sensitive measure of magnetization transfer ratio asymmetry (MTR_asym_) and oxygen-sensitive measure of R_2_’ within the tumor region-of-interest. IDH mutant gliomas showed significantly lower MTR_asym_ × R_2_’ (*p* < 0.001), which differentiated IDH mutation status with sensitivity and specificity of 90.0% and 71.9%. Within IDH mutants, 1p/19q codeletion was associated with lower tumor acidity (*p* < 0.0001, sensitivity 76.9%, specificity 91.3%), while IDH wild-type, EGFR-amplified gliomas were more hypoxic (R_2_’ *p* = 0.024, sensitivity 66.7%, specificity 76.9%). Both R_2_’ and MTR_asym_ × R_2_’ were significantly associated with patient overall survival (R_2_’: *p* = 0.045; MTR_asym_ × R_2_’: *p* = 0.002) and progression-free survival (R_2_’: *p* = 0.010; MTR_asym_ × R_2_’: *p* < 0.0001), independent of patient age, treatment status, and IDH status. The pH- and oxygen-sensitive MRI is a clinically feasible and potentially valuable imaging technique for distinguishing glioma subtypes and providing additional prognostic value to clinical practice.

## 1. Introduction

Metabolic reprogramming in gliomas is a consequence of both genetic alternations and the unique surrounding microenvironment, as well as the interactions between them [1]. The altered metabolic phenotype in turn contributes to the abnormal tumor microenvironment. Hypoxia and tissue acidosis are two important characteristics of the glioma microenvironment [2,3], both associated with a more aggressive phenotype, by regulating multiple biological processes, including invasion, angiogenesis, immunosuppression, chemoresistance, and induction of a glioma stem cell phenotype [2,4,5,6].

Characterization of patient glioma microenvironment could provide valuable information to improve patient diagnosis, prognosis, and treatment assessment, as well as to better understand the pathophysiology. Among the many direct and indirect assessment methods of acidity and hypoxia, magnetic resonance imaging (MRI) is uniquely suitable for glioma evaluation, due to its non-invasive and non-ionizing nature, and the expanding capacity to measure different tissue properties. In this study, we aimed to analyze the diagnostic and prognostic values of glioma microenvironment characteristics, namely tissue acidity and hypoxia, simultaneously investigated by the amine chemical exchange saturation transfer with spin-and-gradient echo echo-planar imaging (CEST-SAGE-EPI) [7]. The amine CEST-SAGE-EPI technique provides pH sensitivity through pH-dependent CEST contrast, and sensitivity to hypoxia through measurement of reversible transverse relaxation rate R_2_’, which is proportional to oxygen extraction fraction [7,8]. This simultaneous pH- and oxygen-sensitive MRI technique has the advantage of clinically feasible scan time (~7.5 min) and does not require an external contrast agent. The technique has already been demonstrated to show significantly different microenvironment characteristics of different tissue types in glioma patients [7,9], correlation with WHO 2016 glioma grade [7], and the potential to assess treatment efficacy of Bevacizumab in recurrent glioblastoma patients [10].

In this study, we aimed to further validate the clinical usefulness of the pH- and oxygen-sensitive MRI to differentiate key glioma genotypes and to predict patient survival. Although other MR-based approaches have been explored for the same purposes, including MR spectroscopy [11], diffusion imaging [12], perfusion imaging [12,13,14,15,16], and amide proton transfer imaging [17], our technique provides the unique sensitivity to tissue acidosis and hypoxia, which may provide complementary information to the existing approaches.

Specifically, the objectives of this retrospective study were (1) to evaluate the pH- and oxygen-sensitive imaging features associated with glioma genotypes, including isocitrate dehydrogenase (IDH) mutation, 1p/19q co-deletion, and epidermal growth factor receptor (EGFR) amplification, (2) to assess the ability of the proposed imaging technique to differentiate different glioma genotypes, and (3) to investigate the association between these imaging biomarkers with patient overall survival (OS) and progression-free survival (PFS).

## 2. Materials and Methods

### 2.1. Patients

In this study, we retrospectively included 159 histologically confirmed adult glioma patients who received advanced metabolic MRI and routine MRI scans between April 2015 and October 2019. Of the 159 patients, 96 were scanned either prior to radiation therapy and/or chemotherapy including temozolomide (*n* = 85), with (*n* = 16) or without (*n* = 69) prior resection surgery, or had been off treatment for more than one year (*n* = 11). The other 63 patients were either on active treatment or recently off treatment at the time of MRI scanning. Detailed patient characteristics are outlined in Table 1.

We collected information on patient age, sex, and mutation status of the glioma (details of the determination process described in the Supplementary Materials). Additionally, we collected the residual OS of patients, defined as the days between the MRI scan and the date of death, as well as the residual PFS, defined as the days between the MRI scan and the date of the next clinically recorded recurrence or death for all patients. This information was collected in January 2021. At the time of data collection, 63 out of the 159 patients have passed away. The other 96 patients were censored either due to loss in follow-up or having survived past the date of data collection. Of the 159 patients, 80 patients have progressed or passed away. This retrospective study was approved by the local institutional review board in accordance with the Helsinki Declaration, and all patients provided informed written consent prior to the study.

### 2.2. MRI Acquisition and Post-Processing

All patients received standardized brain tumor imaging protocol [18], performed on 3T MR scanners (Trio, Prisma, or Skyra, Siemens Healthcare; Erlangen, Germany). In addition, patients received advanced metabolic imaging with either a single-echo CEST-EPI sequence [19] (*n* = 67) or a multi-echo CEST-SAGE-EPI sequence [7] (*n* = 92), prior to contrast agent administration. Of the 159 patients, 150 also received dynamic susceptibility contrast (DSC) perfusion MRI. The scan time of the CEST-SAGE-EPI is 7.5 min. Detailed information on the MRI sequences are provided in the Supplementary Materials.

We performed the image post-processing using MATLAB (Release 2019b, MathWorks, Natick, MA, USA). All resulting maps were registered to the high-resolution post-contrast T_1_-weighted images for subsequent analyses. CEST-SAGE-EPI data was used to calculate the asymmetric magnetization transfer ratio (MTR_asym_) at amine proton resonance frequency (3.0 ppm) as a measure related to tissue acidity, and the reversible transverse relaxation rate R_2_’ as a measurement sensitive to paramagnetic deoxyhemoglobin. We also calculated the relative cerebral blood volume (rCBV) using the DSC-MRI data. Detailed procedures of post-processing are provided in the Supplementary Materials. To summarize, CEST images were processed for all 159 patients; R_2_’ images were available for 92 patients scanned with CEST-SAGE-EPI sequence; and rCBV maps were available for 150 patients.

### 2.3. MRI Features Extraction

**Figure 1:**
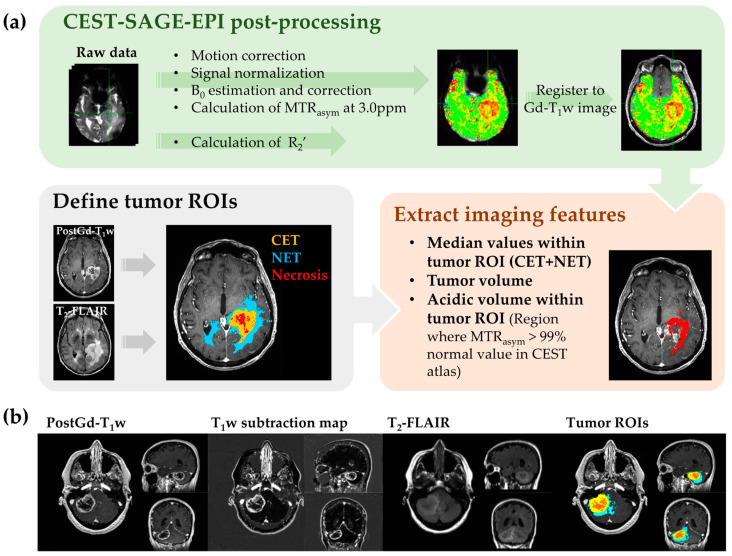
Workflow diagram of CEST-SAGE-EPI data processing and feature extraction. The image post-processing procedure is described in (**a**). (**b**) Demonstrates an example of tumor ROIs delineated semi-automatically using T_1_-weighted subtraction maps and T_2_-weighted FLAIR images, with the yellow ROI representing the contrast-enhancing tumor, red representing the region of central necrosis, and cyan showing the non-enhancing tumor (or edema) region.

We defined three mutually exclusive tumor regions-of-interest (ROIs) using a semi-automated thresholding method as reported previously [20]. The tumor ROIs included: (1) contrast-enhancing tumor (CET) defined by T_1_-weighted subtraction map [20]; (2) regions of central necrosis defined by hypointensity on T_1_-weighted subtraction map within CET; and (3) hyperintense regions on T_2_-weighted FLAIR images, excluding areas of necrosis and contrast enhancement (non-enhancing tumor, NET). An example of tumor ROIs is demonstrated in Figure 1.

**Figure 2:**
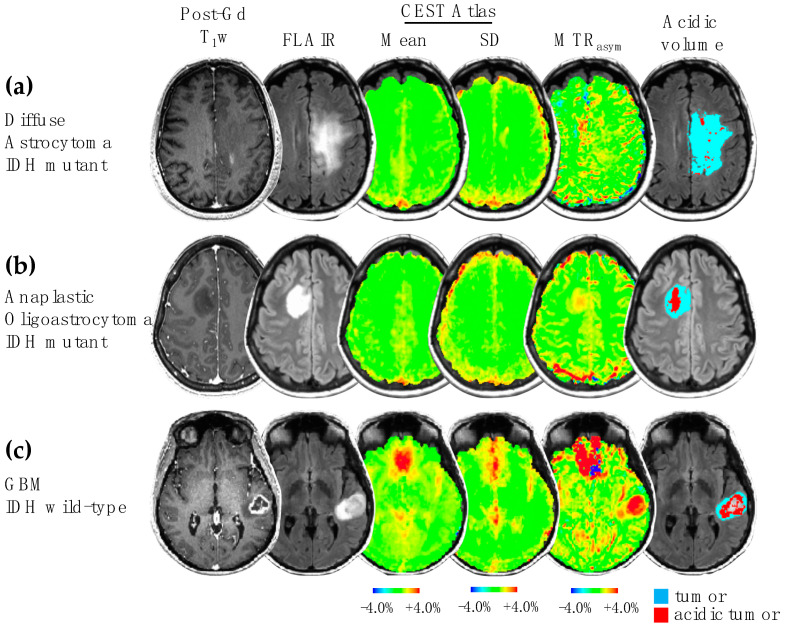
Examples of acidic volume determination with CEST atlas. Three patient examples with glioma grade II (**a**), grade III (**b**), and grade IV (**c**) are demonstrated, showing post-contrast T_1_-weighted image, FLAIR image, CEST atlas, MTR_asym_ at 3.0 ppm, and the acidic volume within the tumor. The high MTR_asym_ in the frontal brain region in patient (**c**) was due to an artifact caused by large B_0_ inhomogeneity in this region. SD: standard deviation.

Four imaging features were extracted from the parametric maps: the median values of MTR_asym_ at 3.0 ppm, R_2_’, rCBV, and the product MTR_asym_ × R_2_’ (reflecting the degree of both acidity and hypoxia) within the tumor ROI excluding necrosis (combined ROI of CET and NET) were calculated for analysis. Additionally, we quantified the volume of tumor ROI, the acidic volume of tumor, and the acidic volume fraction, for use in analyses of association with patient prognosis. The calculation of the acidic tumor volume and the acidic volume fraction is described in the Supplementary Materials. Three patient examples are demonstrated in Figure 2.

### 2.4. Data Analysis and Statistics

We used Student *t*-test or Mann–Whitney U test, based on the normality status of the data, to compare MRI features between different genotypes: IDH mutant versus wild-type, 1p/19q co-deleted versus non-co-deleted, and EGFR-amplified versus non-amplified gliomas. The MRI features were also compared across tumor tissue types using Kruskal–Willis ANOVA. Subsequent multiple comparisons between the groups were performed with the Tukey–Kramer approach. All metrics are reported as mean ± standard deviation. Receiver operating characteristic (ROC) analysis was performed to assess the ability of the MRI features to discriminate different genotypes. The area under the curve (AUC), cut-off value, sensitivity, specificity, and prediction accuracy (percentage of cases predicted correctly) were reported.

We used Cox regression analysis and log-rank comparison of Kaplan–Meyer curves to evaluate the prognostic factors for residual OS and PFS. For Cox regression analysis, we performed both univariate analyses with the clinical variables (age, treatment status, and IDH status) and imaging features, as well as multivariate analyses with the imaging features, using the clinical features as covariates. 1p/19q status and EGFR status were not included as covariates due to the unavailability of the information for some patients and their high correlations with IDH status. The *p*-value, the hazard ratio (HR), and the confidence interval (CI) of the HR were reported. Besides, we have plotted the Kaplan–Meyer curves for patients stratified with different clinical variables and imaging features and performed log-rank analyses to determine the prognostic value of these variables. For binary variables, including IDH status, 1p/19q status, and EGFR status, the patients were stratified according to the presence or absence of the genetic alternation. For continuous variables, including age and all imaging features, the median values were used for separating the patient groups. *p*-values less than 0.05 were considered statistically significant. All calculations and analysis were carried out using MATLAB (Release 2019b, MathWorks, Natick, MA, USA).

## 3. Results

**Figure 3:**
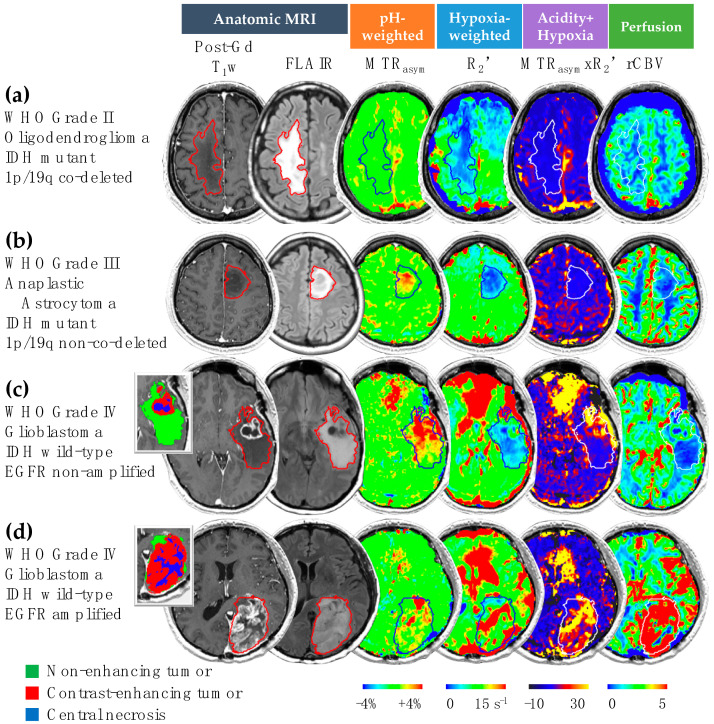
pH- and oxygen-sensitive MR images in representative glioma patients. Four patient examples with IDH mutant 1p/19q co-deleted glioma (**a**), IDH mutant 1p/19q non-co-deleted glioma (**b**), IDH wild-type EGFR non-amplified glioma (**c**), and IDH wild-type EGFR amplified glioma (**d**) are illustrated. Tumors are outlined in each image, with segmented tumor ROIs demonstrated for patients (**c**,**d**). Regions with elevated acidity, high hypoxia, and increased perfusion in the pH-weighted images, hypoxia-sensitive images, and perfusion images are represented by red colors, corresponding to high values of MTR_asym_, R_2_’, and rCBV, respectively. Similarly, a high level of combined acidity and hypoxia is highlighted in yellow on the MTR_asym_ × R_2_’ map.

Four patient examples from different genotypes are demonstrated in Figure 3. In general, we observed that IDH mutant gliomas (Figure 3a,b) had lower acidity and hypoxia compared with IDH wild-type gliomas (Figure 3c,d). The IDH wild-type GBM patient showed high CEST and R_2_’ contrast, especially within the contrast-enhancing areas (Figure 3c,d). The acidic and hypoxia imaging features were significantly different across different tissue types: NAWM, CET region, NET region, and necrosis area. Detailed description the comparisons can be found in the Supplementary Materials.

### 3.1. Acidity and Hypoxia in IDH Mutant and Wild-Type Gliomas

**Figure 4:**
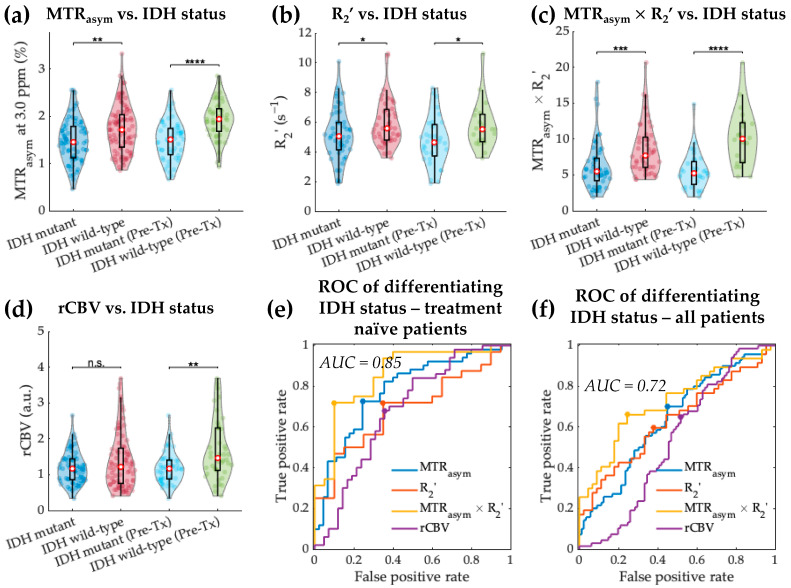
IDH status differentiation based on MTR_asym_, R_2_’, MTR_asym_ × R_2_’, and rCBV. Violin plot graphs of IDH mutant and wild-type gliomas imaging features (**a**–**d**) are demonstrated for both the entire patient cohort and treatment-naïve patients. The red dots represent the median value, while the upper and lower edges of the box plots represent the 25th and 75th percentile values, respectively. The receiver operating characteristic curves for treatment-naïve patient cohort are plotted in (**e**), showing the best differentiation with MTR_asym_ × R_2_’ (AUC = 0.85). ROC curves for all patients are plotted in (**f**). *: *p*-value < 0.05; **: *p*-value < 0.01; ***: *p*-value < 0.001; ****: *p*-value < 0.0001; n.s.: non-significant.

As demonstrated in Figure 4a–c, tumor region MTR_asym_ at 3.0 ppm, R_2_’, and MTR_asym_ × R_2_’ all showed significantly lower values in IDH mutant gliomas compared with IDH wild-type gliomas (MTR_asym_ 1.48 ± 0.45% vs. 1.73 ± 0.50%, *p* = 1.41 × 10^−3^; R_2_’ 5.17 ± 1.74 s^−1^ vs. 5.94 ± 1.54 s^−1^, *p* = 0.030; MTR_asym_ × R_2_’ 6.26 ± 3.41 vs. 8.60 ± 3.57, *p* = 2.57 × 10^−4^). These significant differences remained when considering only the treatment-naïve patients (MTR_asym_ *p* = 1.23 × 10^−6^; R_2_’ *p* = 0.025; MTR_asym_ × R_2_’ *p* = 2.22 × 10^−5^). After excluding GBM, the tumor acidity remained to be significantly lower in IDH mutant gliomas (MTR_asym_ 1.43 ± 0.42% vs. 1.83 ± 0.48%, *p* = 1.44 × 10^−3^), while the tumor hypoxia level reflected by R_2_’ was no longer significantly different between IDH mutant and wild-type gliomas (*p* = 0.744). rCBV showed significantly higher value in IDH wild-type compared with the mutant gliomas in treatment-naïve patients (Figure 4d; IDH mutant 1.19 ± 0.44 vs. IDH wild-type 1.69 ± 0.85, *p* = 0.005) but not in all patients.

ROC analysis suggested the best differentiation of treatment-naïve IDH mutant from wild-type gliomas was achieved using MTR_asym_ × R_2_’ with a threshold of 6.00, which resulted in a sensitivity and specificity of 90.0% and 71.9%, respectively (Figure 4e, AUC 0.85, accuracy 78.8%). This combined acidity and hypoxia metric was also able to differentiate IDH mutation status when considering the entire patient cohort, with slightly lower sensitivity (75.6%) and specificity (66.0%) (Figure 4f, AUC 0.72, accuracy 70.7%).

### 3.2. Acidity and Hypoxia in 1p/19q Co-Deleted and Non-Co-Deleted IDH Mutant Gliomas

**Figure 5:**
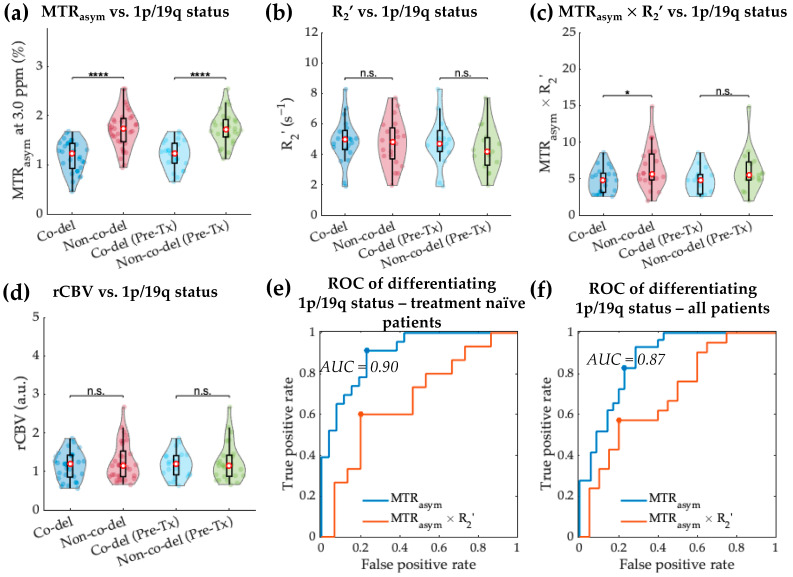
1p/19q co-deletion status differentiation within IDH mutant gliomas based on MTR_asym_, R_2_’, MTR_asym_ × R_2_’, and rCBV. Violin plot graphs of IDH mutant 1p/19q co-deleted and non-col-deleted gliomas imaging features (**a**–**d**) are demonstrated for both the entire patient cohort and treatment-naïve patients. The red dots represent the median value, while the upper and lower edges of the box plots represent the 25th and 75th percentile values, respectively. The receiver operating characteristic curves for treatment-naïve patient cohort are plotted in (**e**), showing the best differentiation with MTR_asym_ (AUC = 0.90). ROC curves for all patients are plotted in (**f**). *: *p*-value < 0.05; ****: *p*-value < 0.0001; n.s.: non-significant.

When comparing between 1p/19q genotypes within the IDH mutant gliomas, MTR_asym_ at 3.0 ppm was significantly lower in 1p/19q co-deleted gliomas (Figure 5a; 1.18 ± 0.31% vs. 1.72 ± 0.39%, *p* = 7.44 × 10^−8^). No significant difference in R_2_’ was observed between the co-deleted and non-co-deleted gliomas (Figure 5b; *p* = 0.792). Similar to the MTR_asym_ measurement, MTR_asym_ × R_2_’ was significantly lower in 1p/19q co-deleted IDH mutant gliomas (Figure 5c, MTR_asym_ × R_2_’ *p* = 0.022), while the difference in MTR_asym_ × R_2_’ was no longer significant when only treatment-naïve patients were considered (MTR_asym_ *p* = 2.02 × 10^−7^, R_2_’ *p* = 0.426, MTR_asym_ × R_2_’ *p* = 0.115). rCBV did not demonstrate significant difference between the two 1p/19q genotypes.

The ROC analyses (Figure 5e,f) showed that the prediction of 1p/19q status in treatment-naïve IDH mutant gliomas was best achieved using MTR_asym_ with a threshold of 1.55%. The differentiation using tumor acidity had a sensitivity of 76.9% and a specificity of 91.3% (AUC 0.90, accuracy 83.7%). The performance of classifying 1p/19q status using MTR_asym_ for all patients was similar, with AUC of 0.87 (threshold 1.46%, sensitivity 77.1%, specificity 82.8%, and accuracy 79.7%).

### 3.3. Acidity and Hypoxia in EGFR-Amplified and Non-Amplified IDH Wild-Type Gliomas

**Figure 6:**
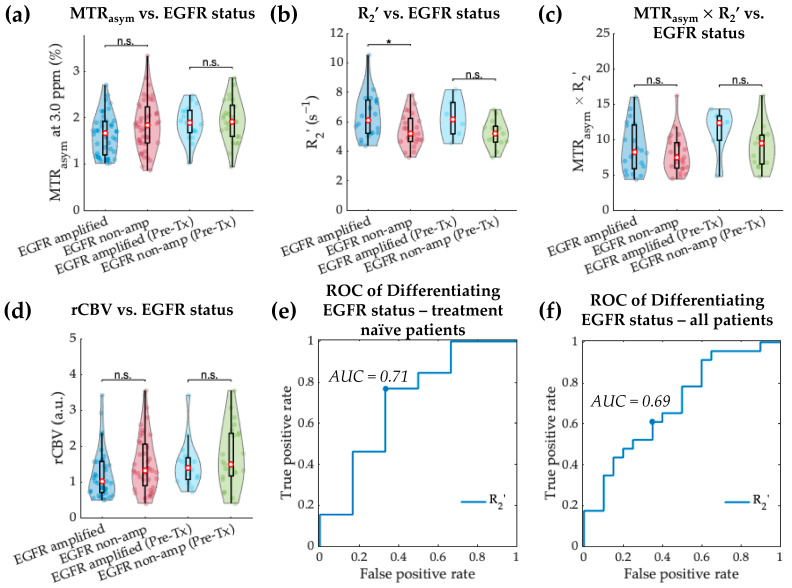
EGFR amplification status differentiation within IDH wild-type gliomas based on MTR_asym_, R_2_’, MTR_asym_ × R_2_’, and rCBV. Violin plot graphs of IDH wild-type EGFR-amplified and non-amplified gliomas imaging features (**a**–**d**) are demonstrated for both the entire patient cohort and treatment-naïve patients. The red dots represent the median value, while the upper and lower edges of the box plots represent the 25th and 75th percentile values, respectively. The receiver operating characteristic curves for treatment-naïve patient cohort are plotted in (**e**), showing the best differentiation with R_2_’ (AUC = 0.71). ROC curves in all patients are plotted in (**f**). *: *p*-value < 0.05; n.s.: non-significant.

MTR_asym_ at 3.0 ppm and MTR_asym_ × R_2_’ were not significantly different between EGFR-amplified and non-amplified tumors (Figure 6a,c; MTR_asym_ *p* = 0.069 for all patients; MTR_asym_ × R_2_’ *p* = 0.503 for all patients). Median R_2_’ was significantly higher in EGFR-amplified than non-amplified gliomas (Figure 6b; 6.36 ± 1.52 s^−1^ vs. 5.41 ± 1.12 s^−1^, *p* = 0.024). The same trend was preserved in treatment-naïve patients with non-significant difference (6.25 ± 1.36 s^−1^ vs. 5.20 ± 0.95 s^−1^, *p* = 0.066), likely due to the small number of subjects in each group (EGFR-amplified *n* = 6, non-amplified *n* = 13). No significant difference was observed in median rCBV between EGFR-non-amplified and amplified gliomas either in the entire patient cohort (Figure 6d; 1.22 ± 0.70 vs. 1.50 ± 0.76, *p* = 0.071) or in treatment-naïve patients (*p* = 0.449).

The ROC analysis showed that R_2_’ was able to differentiate EGFR amplification status in treatment-naïve IDH wild-type gliomas with a threshold of 5.71 s^−1^ (Figure 6e; AUC 0.71, sensitivity 66.7%, specificity 76.9%, accuracy 73.7%). Similar results were obtained when including all patients, with AUC of 0.69 (Figure 6f; threshold 5.56 s^−1^, sensitivity 65.0%, specificity 60.8%, accuracy 69.1%).

### 3.4. Acidity and Hypoxia Imaging Features Correlating with Glioma Patient Survival

Cox regression analysis was used to determine the effect of clinical factors and MRI measurements on residual OS (Table 2). Univariate analyses showed a significant decrease in residual OS associated with elderly patients (*HR* = 1.04, *p* = 1.75 × 10^−4^), being on treatment (*HR* = 3.75, *p* = 3.84 × 10^−7^), IDH wild-type status (*HR* = 0.09, *p* = 4.40 × 10^−9^), higher R_2_’ (*HR* = 1.44, *p* = 2.02 × 10^−4^), higher MTR_asym_ × R_2_’ (*HR* = 1.14, *p* = 7.77 × 10^−4^), larger tumor (*HR* = 1.01, *p* = 7.78 × 10^−5^), larger acidic tumor volume (*HR* = 1.02, *p* = 2.74 × 10^−4^), and higher acidic tumor volume fraction (*HR* = 1.02, *p* = 0.041). When considering age, treatment status, and IDH status as covariates in a multivariable model, R_2_’ and MTR_asym_ × R_2_’ remained to be significantly associated with patient survival (R_2_’: *HR* = 1.27, *p* = 0.045; MTR_asym_ × R_2_’: *HR* = 1.17, *p* = 0.002). Within the treatment-naïve patients, MTR_asym_ at 3.0 ppm also showed significant association with residual OS (Supplementary Materials Table S1; *HR* = 3.72, *p* = 0.003), in addition to R_2_’ and MTR_asym_ × R_2_’ (R_2_’: *HR* = 1.66, *p* = 0.011; MTR_asym_ × R_2_’: *HR* = 1.1.27, *p* = 0.002).

The clinical factors and MRI measurements performed similarly at predicting residual PFS (Table 3). Univariate analyses showed a significant decrease in residual PFS associated with elderly patients, being on treatment, IDH wild-type status, higher R_2_’ (*HR* = 1.44, *p* = 4.59 × 10^−5^), higher MTR_asym_ × R_2_’ (*HR* = 1.18, *p* = 8.29 × 10^−6^), larger tumor (*HR* = 1.01, *p* = 1.90 × 10^−4^), greater acidic tumor volume (*HR* = 1.02, *p* = 1.56 × 10^−4^), and higher acidic tumor volume fraction (*HR* = 1.02, *p* = 0.013). One difference from the residual OS analysis was that the MTR_asym_ at 3.0 ppm was also predictive of residual PFS (*HR* = 1.65, *p* = 0.029). When considering age, treatment status, and IDH status as covariates in a multivariable model, again R_2_’ and MTR_asym_ × R_2_’ remained to be significantly associated with residual PFS (R_2_’: *HR* = 1.30, *p* = 0.010; MTR_asym_ × R_2_’: *HR* = 1.19, *p* = 8.62 × 10^−5^). Within the treatment-naïve patients (Supplementary Materials Table S2), MTR_asym_ at 3.0 ppm showed a significant association with PFS (*HR* = 3.10, *p* = 0.002), although the association was lost after using a multivariable model using age and IDH status as covariates.

**Figure 7:**
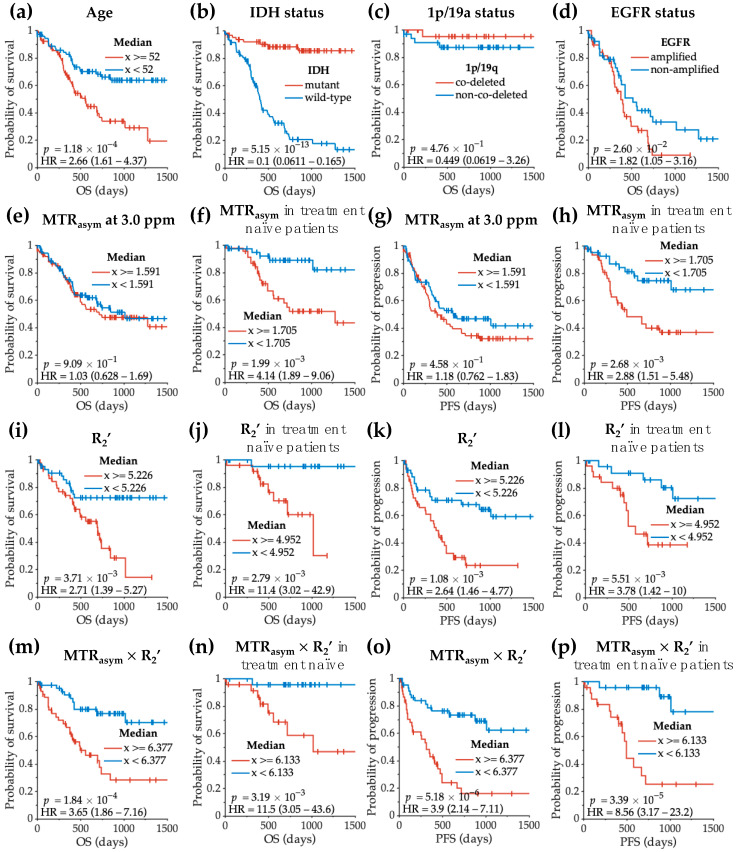
Log-rank analyses of residual overall survival (OS) and residual progression-free survival (PFS) with clinical variables (**a**–**d**) and MRI features (**e**–**p**). The log-rank tests were performed with the entire patient cohort if not otherwise specified. The OS refers to the residual OS from the patient scan date to the date of death. The PFS refers to the residual PFS from patient scan date to the date of disease recurrence.

Log-rank analyses confirmed that residual OS was significantly associated with patient age and genetic alternations (Figure 7a–d). Analysis of residual survival in patients grouped by high or low MTR_asym_ showed no significant difference when examining all patients (Figure 7e, *p* = 0.909); however, a significant survival benefit was observed in treatment-naïve patients with lower acidity (Figure 7f, *p* = 0.002). In both treatment-naïve patients and in all patients, lower R_2_’ provided a significant survival advantage (Figure 7i,j; all patients *p* = 0.004; treatment-naïve patients *p* = 0.003). Similarly, lower MTR_asym_ × R_2_’ was associated with longer residual OS in both treatment-naïve patients (Figure 7n, *p* = 0.003) and in the entire patient cohort (Figure 7m, *p* = 0.0002). The same results were observed for residual PFS (Figure 7g,h,k,l,o,p).

**Figure 8:**
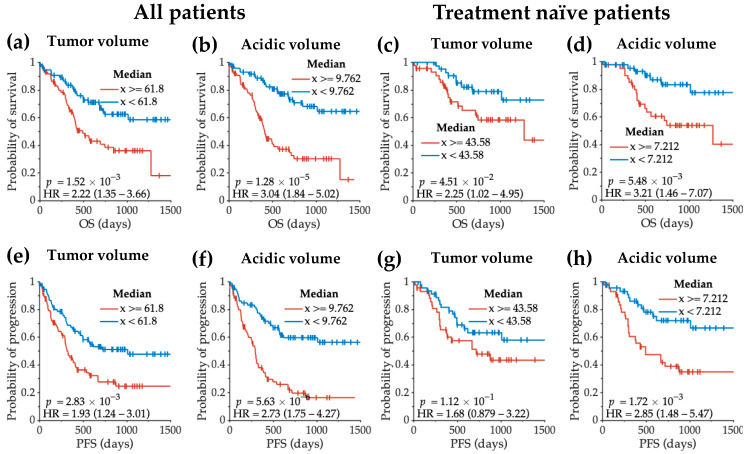
Log-rank analyses of residual overall survival (OS) and residual progression-free survival (PFS) with tumor volume and acidic volume. The log-rank comparisons of OS between low and high tumor volume are plotted in (**a**,**c**), in the entire patient cohort and in treatment-naïve patients only, respectively. Similarly, the log-rank comparisons of OS between low and high acidic tumor volume are plotted in (**b**,**d**). (**e**–**h**) demonstrate the log-rank comparisons of PFS. The OS refers to the residual OS from patient scan date to the date of death. The PFS refers to the residual PFS from patient scan date to the date of disease recurrence.

Tumor volume and acidic tumor volume both stratified patients into significantly different risk groups. Patients with smaller tumors had longer residual overall survival (Figure 8a,c; all patients *p* = 0.002; treatment-naïve patients *p* = 0.045). This result was also observed for patients with smaller acidic tumor volume (Figure 8b,d; all patients *p* = 1.28 × 10^−5^; treatment-naïve patients *p* = 0.006), with even higher levels of significance. For residual PFS, tumor volume also stratifies patient risk in the overall patient cohort (Figure 8e, *p* = 0.003) but not in treatment-naïve patients (Figure 8g, *p* = 0.112). On the other hand, patients with lower acidic tumor volume had significantly longer residual PFS in both the entire patient cohort (Figure 8f, *p* = 5.63 × 10^−6^) and the treatment-naïve patient cohort (Figure 8h, *p* = 0.002).

## 4. Discussion

Taking advantage of the pH sensitivity provided by CEST contrast and sensitivity to deoxyhemoglobin of relaxation rate R_2_’, we are able to simultaneously obtain tumor microenvironment information using the amine CEST-SAGE-EPI technique. The current study validated that the degree of tumor acidity and hypoxia were associated with glioma genotypes, confirming the results from previous studies with smaller sample sizes [9,21,22]. More specifically, IDH mutant gliomas exhibit both lower acidity and lower hypoxia compared with IDH wild-type gliomas. Within the IDH mutant gliomas, 1p/19q co-deletion is associated with lower tumor acidity. Within the IDH wild-type gliomas, EGFR amplification is related to a higher level of hypoxia. In addition, the acidity and hypoxia imaging biomarkers were predictive of patient survival independent of clinical status, indicating that these biomarkers could provide additional value to prognostication.

Molecular biomarkers, together with the histopathological features, provide well-established clinical values of glioma diagnosis and prognostication [23,24,25]. However, these specimen-based approaches require invasive procedures, which largely reduce the clinical practicality of longitudinal monitoring of disease progression. Additionally, sampling bias may affect the accuracy of the assessment and fail to evaluate the intra-tumoral heterogeneity [26,27]. It is thus of great benefit to develop imaging techniques as non-invasive biomarkers for both preoperative disease evaluation and long-term monitoring.

With the combined pH- and oxygen-sensitive imaging biomarker, we were able to differentiate IDH mutation status with high sensitivity (90%) and moderate specificity (72%). Several other non-invasive MR-based approaches have also been shown to differentiate IDH mutation status, including magnetic resonance spectroscopy-based detection of 2-hydroxyglutarate [11], diffusion imaging [12], perfusion imaging [12,13], amide proton transfer-weighted imaging (APT) [17], as well as machine learning and radiomic approaches [28,29]. Our results also demonstrate that amine CEST provides an imaging biomarker for identifying 1p/19q co-deletion, with moderate sensitivity (77%) and high specificity (91%). Previous studies showed that 1p/19q co-deleted gliomas were also characterized with the absence of T_2_-FLAIR mismatch [30], higher rCBV [14], and increased uptake of ^18^F-fluorodeoxyglucose [31]. Deep learning and radiomic methods were also able to differentiate 1p/19q genotypes [29,32]. Lastly, the hypoxia imaging marker R_2_’ performed moderately in differentiating EGFR amplification status, with modest sensitivity (66.7%) and specificity (76.9%). Several studies have predicted EGFR amplification using other imaging characteristics, including a higher maximum rCBV [15]. We did not find a significant difference in median tumor rCBV between the two genotypes, which is consistent with another previous study [16]. The discrepancy of rCBV findings is likely due to the heterogeneous composition of gliomas in which EGFR-amplified and non-amplified tumor cells co-exist. Studies have also reported a lower mean apparent diffusion coefficient using diffusion MRI [16,33], increased ratio of T_2_-bright volume to enclosed T_1_-enhancing volume, and decreased T_2_ border sharpness associated with EGFR amplification [34]. Compared with other imaging techniques, our proposed method has the unique capability of targeting two key components of tumor microenvironment and differentiating IDH mutation, 1p/19q co-deletion, and EGFR amplification simultaneously. Future studies comparing these techniques with the current approach are necessary to understand the correlation between the various physiological parameters.

**Figure 9:**
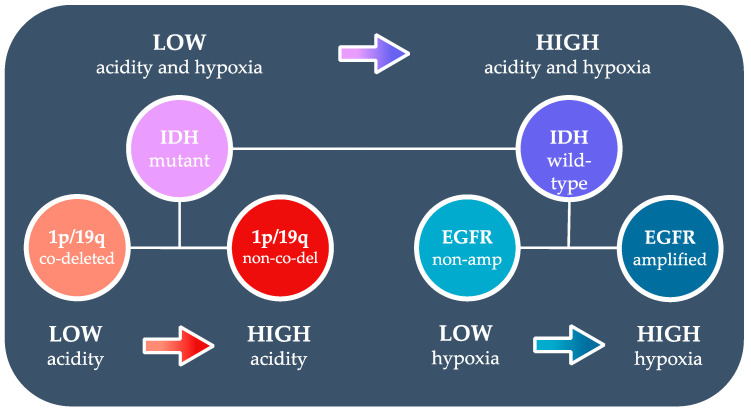
Summary of hypothesized tumor microenvironment differences in glioma genotypes.

In addition to the diagnostic value, the pH- and oxygen-sensitive imaging technique provides new insight into the mechanisms of glioma microenvironment characteristics associated with different genomic alternations (Figure 9). Our observation of lower tissue acidity in IDH mutant gliomas supports previous reports of decreased levels of hypoxia-inducible factor 1 alpha (HIF1α) and HIF1α-responsive genes, including essential genes involved in glycolytic process [35,36]. The investigation within IDH mutant glioma patients further suggested that 1p/19q co-deleted gliomas have lower acidity compared with intact gliomas. Unlike in IDH mutation, the mechanism underlying low extracellular acidity associated with 1p/19q co-deletion is largely unknown. The silencing of sodium-hydrogen exchanger NHE-1 subsequent to IDH-associated DNA hypermethylation and 1p allelic loss [37] could potentially impair the ability of tumor cells to remove intracellular protons, resulting in reduced extracellular acidity. The lower level of acidification in IDH mutant gliomas and 1p/19q co-deleted gliomas revealed by this study is consistent with the less aggressive clinical course and higher sensitivity to therapies in these patients. Although few studies have examined R_2_’ in gliomas, it is conceivable that lower R_2_’ in IDH mutant gliomas could be due to lower proliferation rates and less angiogenesis compared with IDH wild-type gliomas. Within IDH wild-type gliomas, the higher hypoxia associated with EGFR amplification may be explained by two mechanisms: the translational up-regulation of EGFR induced by hypoxic microenvironment [38] and the enhanced oncogenic pathways downstream to increased EGFR activation [39].

Another key finding of the current study is that patients with more acidic and hypoxic tumors were likely to have shorter survival. This is in accordance with the literature showing tumor acidity and hypoxia are related to a more aggressive phenotype. Limited studies have been performed to evaluate the prognostic value of non-invasive biomarkers of tumor microenvironment in human subjects. Spence et al. showed that greater hypoxic volume and higher maximum level of hypoxia measured using ^18^F-fluoromisonidazole PET were associated with shorter OS and PFS [40]. Compared with PET, MRI techniques have the advantage of higher spatial resolution and no dependency on tracer distribution in the brain. Recently, Paech et al. demonstrated that relaxation-compensated APT MRI at 7T was associated with survival in high-grade glioma patients [41]. The current study demonstrated the ability to use 3T MRI biomarkers targeting tumor microenvironment to predict patient outcome.

Additionally, the log-rank analysis showed that the acidic tumor volume exhibited better delineation of patients with short and long residual OS/PFS compared with tumor volume from anatomic imaging, indicating that acidic tumor volume might serve as a better measure of tumor burden. The current criteria of glioma radiographic response rely heavily on the standard anatomic T_1_-weighted and T_2_-weighted images. However, tumor volume evaluated using T_2_/FLAIR images is often unable to differentiate between infiltrative tumor and vasogenic edema. Response assessment that solely relies on anatomic MRI scans may be confounded by treatment-related pseudo-progression and pseudo-response. Advanced MRI biomarkers, including radiomics [42,43] and other imaging biomarkers reflecting the pathophysiological features of glioma, may help to offer a potential solution. Although the current study showed promising risk stratification ability of acidic tumor volume, further validation and evaluation in treatment response assessment are needed to prove its clinical value.

One limitation of this study is the heterogeneous population, with both newly diagnosed, treatment-naïve patients, and patients treated with radiation therapy, chemotherapy, anti-angiogenic drugs, immunotherapy, or other investigative treatment methods. The retrospective nature of the current study is another limitation. Although the current mixed patient cohort could ensure a more generalizable conclusion, future prospective studies with a more clearly defined population, such as recurrent GBM patients treated with standard chemoradiation therapy, may provide greater clinical value.

## 5. Conclusions

The current study suggests simultaneous pH- and oxygen-sensitive amine CEST-SAGE-EPI is a clinically feasible and potentially valuable imaging technique for distinguishing glioma subtypes, revealing unique characteristics associated with IDH mutation, 1p/19q co-deletion, and EGFR amplification. The proposed imaging biomarkers were also predictive of patient residual OS and PFS. Patients with more acidic and hypoxic tumors had significantly shorter survival. These non-invasive imaging biomarkers could provide additional diagnostic and prognostic value to clinical practice.

## Figures and Tables

**Table 1:** Patient demographics.

		WHO 2016 Grading		
	All Patients	Grade II	Grade III	Grade IV
Number of patients (treatment naïve/on treatment)	159 (96/63)	42 (33/9)	38 (28/10)	79 (35/44)
Age median (range)	52 (19–90)	41 (22–90)	48.5 (21–70)	59 (19–83)
Sex male/female	101/58	24/18	24/14	53/26
IDH status wild-type/mutant	89/70	3/39	13/25	73/6
1p/19q status in IDH mutant non-co-deleted/co-deleted/NA	35/29/6	16/20/3	15/9/1	4/0/2
EGFR status in IDH wild-type non-amplified/amplified/NA	42/40/7	2/1/0	9/3/1	31/36/6

**Table 2:** Cox proportional-hazards model analysis of glioma residual overall survival (all patients).

Characteristics	OS (Univariate)			OS (Multivariate)		
	*p*-Value	*HR*	*HR* [95% CI]	*p*-Value	*HR*	*HR* [95% CI]
Age	*** 1.753 × 10^−4^	1.035	1.017–1.054	Covariate		
Treatment status	**** 3.837 × 10^−7^	3.748	2.250–6.241	Covariate		
IDH	**** 4.400 × 10^−9^	0.093	0.042–0.206	Covariate		
MTR_asym_ at 3.0 ppm	0.2280	1.360	0.825–2.242	0.5474	1.1841	0.683–2.053
R_2_’	*** 2.019 × 10^−4^	1.440	1.188–1.746	* 0.0445	1.2703	1.006–1.604
MTR_asym_ × R_2_’	*** 7.767 × 10^−4^	1.140	1.056–1.231	** 0.0019	1.1655	1.058–1.284
rCBV	0.9646	0.991	0.661–1.486	0.9702	0.9924	0.664–1.483
CET + NET volume	**** 7.784 × 10^−5^	1.007	1.003–1.010	0.1526	1.0027	0.999–1.006
Acidic volume	*** 2.742 × 10^−4^	1.020	1.009–1.031	0.0931	1.0103	0.998–1.023
Acidic volume fraction	* 0.0410	1.021	1.001–1.041	0.3563	1.0098	0.989–1.031

OS, overall survival; CI, confidence interval; HR, hazard ratio; *: *p*-value < 0.05; **: *p*-value < 0.01; ***: *p*-value < 0.001; ****: *p*-value < 0.0001.

**Table 3:** Cox proportional-hazards model analysis of glioma residual progression-free survival (all patients).

Characteristics	PFS (Univariate)			PFS (Multivariate)		
	*p*-Value	*HR*	*HR* [95% CI]	*p*-Value	*HR*	*HR* [95% CI]
Age	** 0.0014	1.025	1.010–1.041	Covariate		
Treatment status	**** 5.032 × 10^−7^	3.142	2.010–4.910	Covariate		
IDH	**** 4.788 × 10^−11^	0.138	0.077–0.249	Covariate		
MTR_asym_ at 3.0 ppm	* 0.0287	1.653	1.054–2.594	0.1661	1.4278	0.862–2.364
R2’	**** 4.587 × 10^−5^	1.436	1.207–1.709	* 0.0102	1.2972	1.064–1.582
MTR_asym_ × R_2_’	**** 8.290 × 10^−6^	1.177	1.096–1.265	**** 8.617 × 10^−5^	1.1919	1.092–1.301
rCBV	0.2076	1.244	0.886–1.747	0.4957	1.1293	0.796–1.602
CET + NET volume	*** 1.903 × 10^−4^	1.006	1.003–1.009	0.5049	1.0011	0.998–1.004
Acidic volume	*** 1.563 × 10^−4^	1.019	1.009–1.030	0.1072	1.0094	0.998–1.021
Acidic volume fraction	* 0.0125	1.022	1.005–1.040	0.2023	1.0120	0.994–1.031

PFS, progression-free survival; CI, confidence interval; HR, hazard ratio; *: *p*-value < 0.05; **: *p*-value < 0.01; ***: *p*-value < 0.001; ****: *p*-value < 0.0001.

## Data Availability

The data presented in this study are available on request from the corresponding author. The imaging data in this study are not openly available due to HIPAA compliance.

## Supplementary Materials

The following supporting information can be downloaded at: https://www.mdpi.com/article/10.3390/cancers14102520/s1, Supplementary Methods; Figure S1: Tissue type differentiation based on MRI features; Table S1: Cox proportional-hazards model analysis of glioma patient residual overall survival using clinical variables and MRI features (treatment-naïve patients); Table S2: Cox proportional-hazards model analysis of glioma patient residual progression-free survival using clinical variables and MRI features (treatment-naïve patients) [7,18,19,44,45,46,47].
