# Supplementary material for: Diagnostic and Prognostic Value of pH- and Oxygen-Sensitive Magnetic Resonance Imaging in Glioma: A Retrospective Study
Source: Cancers (Basel). 2022 May 20;14(10):2520. doi: 10.3390/cancers14102520 (PMC9139712; doi:10.3390/cancers14102520)
Supplement: Supplementary file 1 [file cancers-14-02520-s001.zip › cancers-1720715-supplementary.pdf]

Methods

Patients

The IDH mutation status was determined by genomic sequencing analysis using the polymerase chain reaction and/or immunohistochemistry as described previously [1] for all patients, using resected or biopsied tissue. 1p/19q loss and EGFR amplification status were assessed with fluorescence in situ hybridization method or obtained from Foundation Medicine. Clinically, elderly patients with GBM were often tested for only IDH1 mutation but not for IDH2 mutation, due to the rare prevalence. Here in this study, we consider all patients with determined mutation in IDH1 (n = 66), IDH1 wild-type with IDH2 mutation (n = 2), and IDH1 wild-type with unknown IDH2 status and 1p/19q co-deletion (n = 2) as IDH mutant glioma patients.

MRI Acquisition

Following the standardized brain tumor imaging protocol [2], anatomic MRI scans consisted of at least T_2-weighted turbo spin-echo images, fluid-attenuated inversion recovery (FLAIR) images (3 mm slice thickness with no interslice gap), and T_1-weighted pre- and post-contrast images (2D axial turbo spin echo with 3 mm slice thickness and no interslice gap or 3D inversion-prepared gradient-echo images with MPRAGE of 1 mm isotropic voxel size).

The amine CEST sequences were composed of a non-selective saturation pulse train of three 100 ms Gaussian pulses, with a peak amplitude of 6 μT and an inter-pulse delay of 5 ms, followed by spoiling gradients before each EPI readout. A total of 29 z-spectral points was acquired, densely sampled around the amine proton resonance frequency (+3.0 ppm), the reference frequency (−3.0 ppm), and the water resonance frequency (0 ppm). We additionally performed a reference (S_0) scan with 4 averages using identical sequence parameters and no saturation pulse. The detailed acquisition parameters were described in previously published works [3,4].

For dynamic susceptibility contrast (DSC) perfusion MRI, patients were administered with a total dose of 0.1 mmol/kg of a gadolinium contrast agent, either gadopentetate dimeglumine (Gd-DTPA; Magnevist, Bayer Schering Pharma, Leverkusen, Germany) or gadobenate dimeglumine (Gd-BOPTA; Multihance, Bracco Diagnostics, Princeton, NJ). Patients were first given a preloaded dose of 0.025 mmol/kg of gadolinium contrast agent to mitigate contrast agent leakage effects. Following at least two minutes of incubation time, 10 to 25 baseline acquisitions were taken. Next, an additional 10-20 ml (0.075 mmol/kg) at a rate of 3-5 ml/second of contrast agent was delivered for dynamic bolus administration. DSC imaging acquisition parameters included: TE = 21.0–23.0 ms, TR = 1250–1290 ms, flip angle = 60°, field of view (FOV) = 240 × 240 mm, matrix size = 128 × 128, slice thickness = 5.0mm with no inter-slice gap, and number of time points = 120. The total scan time for DSC-MRI was between 2 and 3 minutes.

Post-Processing of MRI Data

CEST data obtained from CEST-EPI and CEST-SAGE-EPI sequences were processed with the following steps: (1) motion correction using affine transformation (*mcflirt*; Functional Magnetic Resonance Imaging of the Brain Software Library, Oxford, United Kingdom); (2) B_0 inhomogeneity correction using a z-spectra-based k-means clustering and Lorentzian fitting algorithm [5]; (3) calculation of the asymmetric magnetization transfer ratio (MTR_{asym}) at amine proton resonance frequency as a measure related to tissue acidity. MTR_{asym} at 3.0 ppm was defined using the equation: $MTR_{asym}(3.0\ ppm) = S(-3.0\ ppm)/S_0 - S(+3.0\ ppm)/S_0$, where $S(\omega)$ is the water MR signal available following the saturation pulses with offset frequency ω and S_0 is the reference signal acquired without RF saturation. An integral of width of 0.4 ppm was quantified around both −3.0 and +3.0 ppm −3.2 to −2.8 ppm and +2.8 to +3.2 ppm, respectively) spectral points, in order to improve signal-to-noise ratio (SNR). For CEST-SAGE-EPI data, the mean MTR_{asym} at 3.0 ppm was calculated by averaging the first and second gradient echoes to further enhance the SNR.

For patients who received CEST-SAGE-EPI scans (n = 92), estimates of transverse relaxation rates were obtained using the spin and gradient echo data from the reference images, by solving a system of Bloch equations as described previously [4,6].The R_2 and R_2^* values were estimated for each voxel to create R_2 and R_2^*maps, which were then used to calculate the R_2' map ($R_2' = R_2^* - R_2$), as a measurement sensitive to paramagnetic deoxyhemoglobin.

Using the DSC-MRI data, calculation of cerebral blood volume (CBV) was performed by first motion correcting the dynamic time series (*mcflirt*), followed by a bi-directional contrast agent leakage correction algorithm [7]. The relative CBV (rCBV) maps were then obtained by normalizing the CBV maps with the average CBV value within a 5 mm sphere in the contralateral, normal-appearing white matter (NAWM) region.

MRI Feature Extraction

We used the CEST scans from 20 healthy volunteers (age range 19–64, median age 26, male $n = 10$, female $n = 10$) to create the normal distribution map for each brain voxel, which we refer to as the CEST atlas. All healthy volunteers were scanned with the anatomic MRI and CEST-SAGE-EPI sequence on a 3T Prisma scanner. The MTR_{asym} at 3.0 ppm maps were then registered to the standard brain MNI space using an affine transformation (*flirt*; FSL, Oxford, United Kingdom). The mean and standard deviation (SD) maps were calculated from the 20 registered MTR_{asym} maps. The 99th percentile of normal CEST contrast distribution was calculated as mean + 2.326 × SD for each voxel. 2.326 is the z critical value for one tail α of 0.01. For calculation of acidic tumor volume, the CEST atlas (mean and SD maps) was first registered to the patient space for each patient. Then, we calculated the acidic tumor volume as the volume of the tumor ROI with MTR_{asym} at 3.0 ppm values higher than 99th percentile of normal distribution.

Results

Acidity and Hypoxia in Different Tissue Types

CET region, NET region, and areas of central necrosis exhibited significantly higher acidity compared to NAWM, as measured by MTR_{asym} at 3.0 ppm (**Figure S1(a)**; $p = 3.71 \times 10^{-46}$). The results were consistent in both treatment-naïve patients ($n = 96$) and all patients including patients previously treated with surgical resection with or without radiation and/or chemotherapy ($n = 159$). Areas of necrosis had the highest levels of acidity, followed by regions of CET, NET, and NAWM.

In the subset of patients who received CEST-SAGE-EPI for which R_2' was available ($n = 92$), NET regions (5.38 ± 1.55 s^{-1}) exhibited significantly lower R_2' compared to NAWM (7.35 ± 1.67 s^{-1}, $p = 4.28 \times 10^{-9}$), while CET lesions and necrosis (9.23 ± 4.11 s^{-1}, 8.84 ± 5.34 s^{-1}) exhibited significantly higher R_2' compared to NET lesions (**Figure S1(b)**). No difference in R_2' was observed between CET and necrotic regions ($p = 0.791$). These same trends were observed when examining treatment-naïve patients exclusively ($n = 52$).

The degree of both acidity and hypoxia, quantified by $MTR_{asym} \times R_2'$, followed trends similar to MTR_{asym} at 3.0 ppm (**Figure S1(c)**). Necrosis and CET had the highest $MTR_{asym} \times R_2'$ and were not significantly different (19.15 ± 8.85 vs. 22.58 ± 14.64). CET $MTR_{asym} \times R_2'$ was significantly higher compared with both NET regions (6.61 ± 2.90; $p = 3.77 \times 10^{-9}$) and NAWM (4.21 ± 2.03; $p = 3.77 \times 10^{-9}$). Similar trends were observed when only considering treatment-naïve patients ($n = 52$). We also evaluated tumor perfusion using rCBV. rCBV was significantly higher in CET compared to any other tissue types (**Figure S1(d)**).

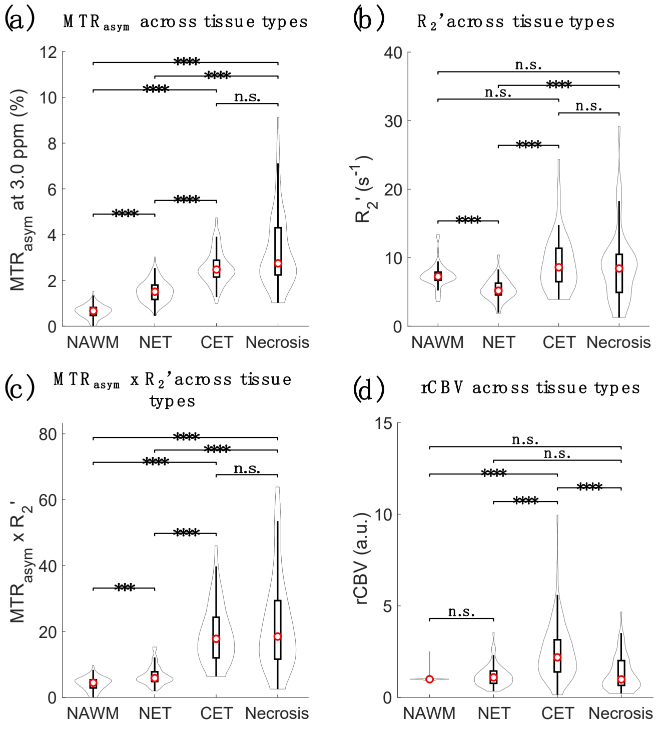

Figure S1. Tissue type differentiation based on MTR_{asym}, R_2', $MTR_{asym} \times R_2'$, and rCBV. Violin plot graphs are demonstrated for imaging features in four mutually exclusive tissue types, including normal appearing white matter (NAWM), T_2-FLAIR hyperintense regions (NET), contrast-enhancing region (CET), and necrosis. The red dots represent the median values, while the upper and lower

edges of the box plots represent the 25th and 75th percentile values, respectively. ***: p-value < 0.001; ****: p-value < 0.0001; n.s.: non-significant.

Acidity and Hypoxia Imaging Features Correlating with Glioma Patient Survival

Table S1. Cox proportional-hazards model analysis of glioma patient residual overall survival using clinical variables and MRI features (treatment-naïve patients).

Characteristics	OS (Univariate)			OS (Multivariate)		
	p-value	HR	HR [95%CI]	p-value	HR	HR [95%CI]
Age	** 0.0023	1.044	1.015–1.073		Covariate	
IDH	*** 2.378×10^{-4}	0.023	0.003–0.173		Covariate	
MTR_{asym} at 3.0 ppm	** 0.0029	3.719	1.566–8.830	0.8911	1.0653	0.431–2.633
R_2'	* 0.0107	1.655	1.124–2.437	* 0.0349	1.7177	1.039–2.840
$MTR_{asym} \times R_2'$	** 0.0016	1.266	1.094–1.465	0.1070	1.1855	0.964–1.458
rCBV	0.0638	1.608	0.973–2.657	0.6581	0.8892	0.529–1.496
CET+NET volume	0.6679	1.002	0.995–1.008	0.3241	0.9965	0.990–1.003
Acidic volume	0.1676	1.016	0.993–1.039	0.7107	0.9951	0.969–1.021
Acidic volume fraction	** 0.0013	1.052	1.020–1.085	0.2237	1.0205	0.988–1.054

OS: overall survival; CI: confidence interval; *: p-value < 0.05; **: p-value < 0.01; ***: p-value < 0.001.

Table S2. Cox proportional-hazards model analysis of glioma patient residual progression-free survival using clinical variables and MRI features (treatment-naïve patients).

Characteristics	PFS (Univariate)			PFS (Multivariate)		
	p-value	HR	HR [95% CI]	p-value	HR	HR [95% CI]
Age	** 0.0085	1.029	1.007–1.051		Covariate	
IDH	**** 7.281×10^{-8}	0.088	0.036–0.212		Covariate	
MTR_{asym} at 3.0 ppm	** 0.0018	3.095	1.523–6.288	0.9024	1.0477	0.498–2.205
R_2'	** 0.0024	1.613	1.185–2.196	** 0.0026	1.9476	1.263–3.004
$MTR_{asym} \times R_2'$	**** 1.705×10^{-5}	1.324	1.165–1.504	** 0.0078	1.2790	1.067–1.533
rCBV	*** 5.535×10^{-4}	2.089	1.375–3.174	0.4432	1.1913	0.761–1.864
CET+NET volume	0.5315	1.002	0.996–1.007	0.1963	0.9964	0.991–1.002
Acidic volume	0.1269	1.014	0.996–1.032	0.6285	0.9950	0.975–1.015
Acidic volume fraction	** 0.0033	1.037	1.012–1.063	0.2237	1.0205	0.988–1.054

PFS: progression-free survival; CI: confidence interval; **: p-value < 0.01; ***: p-value < 0.001; ****: p-value < 0.0001.

References

1. Lai, A.; Kharbanda, S.; Pope, W.B.; Tran, A.; Solis, O.E.; Peale, F.; Forrest, W.F.; Pujara, K.; Lai, J.A.A.; Kharbanda, S.; et al. Evidence for sequenced molecular evolution of IDH1 mutant glioblastoma from a distinct cell of origin. *J Clin Oncol*. **2011**, *29*, 4482–90. doi:10.1200/JCO.2010.33.8715
2. Ellingson, B.M.; Bendszus, M.; Boxerman, J.; Barboriak, D.; Erickson, B.J.; Smits, M.; Nelson, S.J.; Gerstner, E.; Alexander, B.; Goldmacher, G.; et al. Consensus Recommendations for a Standardized Brain Tumor Imaging Protocol in Clinical Trials. *Neuro-Oncol.* **2015**, *17*, 1188–1198. https://doi.org/10.1093/neuonc/nov095.
3. Harris, R.J.; Cloughesy, T.F.; Liau, L.M.; Nghiemphu, P.L.; Lai, A.; Pope, W.B.; Ellingson, B.M. Simulation, Phantom Validation, and Clinical Evaluation of Fast PH-Weighted Molecular Imaging Using Amine Chemical Exchange Saturation Transfer Echo Planar Imaging (CEST-EPI) in Glioma at 3 T. *NMR Biomed.* **2016**, *29*, 1563–1576. https://doi.org/10.1002/nbm.3611.
4. Harris, R.J.; Yao, J.; Chakhoyan, A.; Raymond, C.; Leu, K.; Liau, L.M.; Nghiemphu, P.L.; Lai, A.; Salamon, N.; Pope, W.B.; et al. Simultaneous PH-Sensitive and Oxygen-Sensitive MRI of Human Gliomas at 3 T Using Multi-Echo Amine Proton Chemical Exchange Saturation Transfer Spin-and-Gradient Echo Echo-Planar Imaging (CEST-SAGE-EPI). *Magn. Reson. Med.* **2018**, *80*, 1962–1978. https://doi.org/10.1002/mrm.27204.
5. Yao, J.; Ruan, D.; Raymond, C.; Liau, L.M.; Salamon, N.; Pope, W.B.; Nghiemphu, P.L.; Lai, A; Cloughesy, T.F.; Ellingson, B.M. Improving B0 Correction for pH-Weighted Amine Proton Chemical Exchange Saturation Transfer (CEST) Imaging by Use of k-Means Clustering and Lorentzian Estimation. *Tomography*. **2018**, *4*, 123–137. doi:10.18383/j.tom.2018.00017
6. Schmiedeskamp, H.; Straka, M.; Newbould, R.D.; Zaharchuk, G.; Andre, J.B.; Olivot, J.M.; Moseley, M.E.; Albers, G.W.; Bammer, R. Combined spin- and gradient-echo perfusion-weighted imaging. *Magn Reson Med*. **2012**, *68*, 30–40. doi:10.1002/mrm.23195

7. Leu, K.; Boxerman, J.L.; Lai, A.; Nghiemphu, P.L.; Pope, W.B.; Cloughesy, T.F.; Ellingson, B.M. Bidirectional Contrast agent leakage correction of dynamic susceptibility contrast (DSC)-MRI improves cerebral blood volume estimation and survival prediction in recurrent glioblastoma treated with bevacizumab. *J Magn Reson Imaging*. **2016**, *44*, 1229–1237. doi:10.1002/jmri.25227
